# Anxiety, depression, and motivation for smoking cessation in hospitalized patients with and without cancer

**DOI:** 10.1590/S1806-37132015000100013

**Published:** 2015

**Authors:** Igor Bastos Polônio, Meiryelle Landim Franco, Marina Angélica Mendes Tegon, Célia Beatriz Gianotti Antoneli

**Affiliations:** Irmandade da Santa Casa de Misericórdia de São Paulo, Department of Pulmonology, São Paulo, Brazil, Santa Casa de São Paulo School of Medical Sciences and Professor of Medical Practice, Anhembi Morumbi University; Head, Department of Pulmonology, Irmandade da Santa Casa de Misericórdia de São Paulo, São Paulo, Brazil; Anhembi Morumbi University, School of Medicine, São Paulo, Brazil, Anhembi Morumbi University School of Medicine, São Paulo, Brazil; Anhembi Morumbi University, School of Medicine, São Paulo, Brazil, Anhembi Morumbi University School of Medicine, São Paulo, Brazil; University of Santo Amaro, School of Medicine, São Paulo, Brazil, Anhembi Morumbi University School of Medicine and University of Santo Amaro School of Medicine, São Paulo, Brazil

To the Editor,

We read with interest the article by Almeida et al.^(^ 1 ^)^ published in the Brazilian Journal of Pulmonology. The article addressed smoking habits and nicotine dependence in patients with head and neck cancer. Most of the patients showed high or very high nicotine dependence, and patients with advanced cancer smoked more cigarettes per day than did those with initial cancer, a finding that is paradoxical. However, this reveals the behavior of that specific population, despite their severe disease.

The importance of that finding is that smoking is a chronic disease characterized by nicotine dependence and, therefore, it is included in the International Classification of Diseases.^(^ 2 ^)^ Smoking is one of the major risk factors for the development of various types of cancer. It is estimated that approximately 30% of all malignant tumors are associated with tobacco consumption. In patients with a diagnosis of cancer, smoking accounts for poor response to treatment, decreased survival and quality of life, toxicity to treatment, increased cancer recurrence, and the appearance of metastases. Smoking cessation treatment in this population is extremely difficult, because this population requires specific counseling, psychotherapy, and behavioral intervention, given that recurrence of smoking is very high and pharmacological treatment is not sufficient to maintain patient abstinence for long periods.^(^ 3 ^)^ Consequently, knowing the profile of smokers with cancer is essential to the development of specific strategies for smoking cessation in this population. For this reason, we conducted a prospective observational study involving 50 individuals (smokers, former smokers, and nonsmokers) hospitalized in the clinical medicine ward of a tertiary hospital in the city of São Paulo, Brazil, between February and May of 2014. To that end, the following instruments were administered: the Fagerström Test for Nicotine Dependence^(^ 4 ^)^; the Prochaska & DiClemente Stages of Change Model^(^ 5 ^)^; and the Hospital Anxiety and Depression Scale.^(^ 6 ^)^ The study was approved by the Human Research Ethics Committee of the Anhembi Morumbi University, located in the city of São Paulo. Of the 50 respondents, 18 (36%) reported being nonsmokers, 15 (30%) reported being former smokers, and 17 (34%) reported being smokers. Therefore, most of the individuals analyzed have or have had tobacco exposure. Regarding the sociodemographic profile of the participants, we found that most were male and married and had a low level of education. As for age at smoking initiation, the present study corroborates the findings of previous studies,^(^ 7 ^)^ showing that smoking initiation occurs during adolescence, with 58.3% of smokers having started smoking before the age of 18 years, which indicates the need for campaigns, specifically targeted at this age group, to raise awareness of the harmful effects of smoking among adolescents and young adults. One ethnographic study^(^ 8 ^)^ reported that health concerns are the major motivating factor for smoking cessation. Most of the former smokers (72%) in the present study reported having quit smoking of their own volition, regardless of health problems, which suggests that further research on the subject is needed.

According to Table 1, the sample was homogeneous with regard to gender and age, without statistically significant differences. For statistical analysis, we chose to group smokers and former smokers together, given that former smokers remain at an increased risk for cancer for several years after smoking cessation. In the groups with and without cancer, there were 7 and 8 smokers, respectively. Most of the individuals showed moderate dependence according to the Fagerström Test for Nicotine Dependence^(^ 4 ^)^ (70.6% of the smokers), and none showed high dependence. There were no significant differences in terms of anxiety or depression between the two groups analyzed. We expected that, in the patients with cancer, the levels of anxiety and depression would be higher^(^ 9 ^)^; however, many were in the end stage of the disease and were probably resigned. All of the patients with cancer knew their diagnosis. Regarding anxiety, as determined by the Hospital Anxiety and Depression Scale^(^ 6 ^)^, 83% of the nonsmokers had scores indicating possible anxiety, whereas among smokers and former smokers, scores indicating unlikely anxiety were obtained in 65.6% of the cases. The statistical association was significant, suggesting that nonsmokers were possibly more anxious. Anxiety and depression are known to be greater in smokers.^(^ 2 ^,^ 10 ^)^ Our results could be explained by the possible use of antidepressant and anxiolytic medications by the population of smokers and former smokers. The scale also addresses issues related to depression. In our sample, most of the nonsmokers (72%) and smokers (94%) had scores indicating unlikely depression.

**Table 1 -:** Demographic data as well as data for the Hospital Anxiety and Depression Scale and the Fagerström Test for Nicotine Dependence.

Variable	Smokers and former smokers (n = 31)				p*
	With cancer (n = 19)		Without cancer (n = 12)		
	Mean (SD)	Median (range)	Mean (SD)	Median (range)	
Age	59.8 (9.8)	59 (34-80)	59.8 (17.8)	61.5 (25-81)	0.623
M/F^a^	12/7	(63.2/36.8)	11/1	(91.7/8.3)	0.086^†^
Anxiety	7.2 (4.0)	7.0 (2-15)	4.9 (2.4)	4.0 (2-10)	0.111
Depression	3.9 (3.5)	2.0 (0-13)	2.9 (2.0)	3.0 (0-6)	0.609
Fagerström	4.8 (2.0)	6.0 (1-7)	5.2 (1.5)	5.0 (3-7)	0.804

M/F: male/female. aValues expressed as n/n (%/%).

* Mann-Whitney test.

† Fisher's exact test.

According to Table 2, 86.6% of the smokers were in the precontemplation stage of change, whereas 13.4% were in the contemplation stage of change. Among those with cancer, 6 and 1 were in the precontemplation and contemplation stages of change, respectively. Among those without cancer, all were in the precontemplation stage of change. This finding is very interesting, given that these patients, many of whom had severe disease, were not thinking about quitting smoking (precontemplation stage of change). This shows the importance of the approach of physicians during hospitalization, providing clear information about the risks of smoking and offering specific treatment. It also indicates the need for public health interventions specifically targeted at this population.

**Table 2 -:** Stages of change in 15 smokers as per the Prochaska & DiClemente Stages of Change Model.

Stage of change	Smokers	
	n	%
Precontemplation	13	86.6%
Contemplation	2	13.4%
